# Highly tough and elastic microspheric gel for transarterial catheter embolization in treatment of liver metastasis tumor

**DOI:** 10.1093/rb/rbad026

**Published:** 2023-03-21

**Authors:** Shuyun Wang, Hongjie Yu, Guangsheng Wan, Haowei Fang, Jinxia Mi, Wenqian Xu, Kexiang Sun, Kunxi Zhang, Jingbo Yin, Wanli Deng

**Affiliations:** Department of Medical Oncology, Putuo Hospital, Shanghai University of Traditional Chinese Medicine, Shanghai 200062, P.R. China; Department of Medical Oncology, Putuo Hospital, Shanghai University of Traditional Chinese Medicine, Shanghai 200062, P.R. China; Department of Medical Oncology, Putuo Hospital, Shanghai University of Traditional Chinese Medicine, Shanghai 200062, P.R. China; Department of Polymer Materials, School of Materials Science and Engineering, Shanghai University, Shanghai 200444, P.R. China; Research Center for Health and Nutrition, School of Public Health, Shanghai University of Traditional Chinese Medicine, Shanghai 201203, P.R. China; Department of Medical Oncology, Putuo Hospital, Shanghai University of Traditional Chinese Medicine, Shanghai 200062, P.R. China; Department of Medical Oncology, Putuo Hospital, Shanghai University of Traditional Chinese Medicine, Shanghai 200062, P.R. China; Department of Medical Oncology, Putuo Hospital, Shanghai University of Traditional Chinese Medicine, Shanghai 200062, P.R. China; Department of Polymer Materials, School of Materials Science and Engineering, Shanghai University, Shanghai 200444, P.R. China; Interventional Cancer Institute of Chinese Integrative Medicine, Putuo Hospital, Shanghai University of Traditional Chinese Medicine, Shanghai 200062, P.R. China; Department of Polymer Materials, School of Materials Science and Engineering, Shanghai University, Shanghai 200444, P.R. China; Department of Medical Oncology, Putuo Hospital, Shanghai University of Traditional Chinese Medicine, Shanghai 200062, P.R. China

**Keywords:** microgel, micelle, toughness, liver metastasis, embolization

## Abstract

Transarterial embolization is a widely recognized clinical treatment method for liver tumors. Given that the soft and easily damaged features of embolic particles may limit tumor embolization efficiency, the present study carries out an attempt of fabricating tough and elastic microspheric gel for promoting embolization efficiency. To promote the toughness of hydrogel, poly(ethylene glycol)-*co*-poly(ε-caprolactone)-*co*-poly(ethylene glycol) (PPP) and PPP with two terminal double bonds (PPPDA) are co-assembled into nano-micelles, which are connected with methacrylated chitosan (CSMA) to fabricate microspheric gels via microfluidic technology. Lowering double bond density of micelles promotes the freedom degree of micelles, significantly enhancing hydrogel toughness. To compensate for the strength loss caused by the decrease of double bond density of micelles, phytic acid (PA) are employed to interact with CS to form a physical network, further improving hydrogel strength and toughness. The CS-PPPDA&PPP-PA microspheric gels exhibit higher blocking effect *in vitro*. A rabbit VX2 liver metastasis tumor model is prepared to verify the embolization efficacy of CS-PPPDA&PPP-PA microspheric gels. Compared with clinical used microspheres, fewer CS-PPPDA&PPP-PA microspheric gels can achieve enough embolization efficiency. After embolization for 14 days, CS-PPPDA&PPP-PA microspheric gels exhibit improved tumor necrosis rate and promoted tumor cells apoptosis with reduced inflammation in surrounding tissues, confirming advanced embolic efficiency of tough microgels.

## Graphical Abstract

**Figure:**
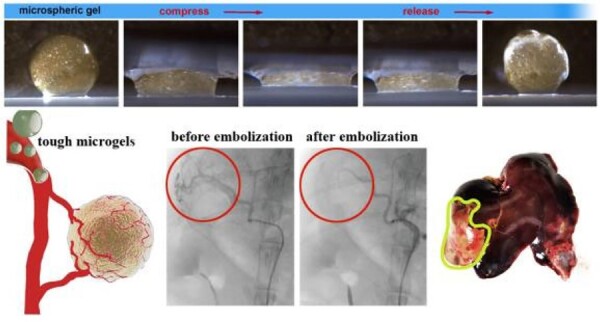
Nanomicelles and biogenic molecule toughening microspheric gel for promoting embolization efficiency.

## Introduction

Liver metastasis (LM) tumor are common malignant tumor of the liver that seriously impact the quality and duration of patient survival [1]. Among the commonly used treatment methods [2, 3], transarterial embolization (TAE) that involves the introduction of an embolic agent into the blood vessel to achieve localized occlusion of blood flow is an invasive technique widely applied in clinic. It is known that vascularization of hepatocellular carcinoma is mostly dependent on the hepatic artery. With the assistance of digital subtraction angiography (DSA), embolic agents such as microspheres are injected into the hepatic artery to block the blood supply to tumors, control tumor growth, and shrink tumors via ischemia and hypoxia [4–6]. While the blood supply to normal liver tissue is maintained by dominant blood flow from the portal vein, thus minimizing damage to the liver.

There are two kinds of microparticles (microspheres) that have been already applied in clinic, including non-degradable embolic agents and degradable ones, both of which are fabricated from polymers [7]. Given that non-degradable embolic agents that fabricated from acrylic polymers and polyvinyl alcohol (PVA) may cause liver tissue infarction at non-tumor sites or causes gallbladder infarction, as well as adverse reactions in patients, embolic agents fabricated from bio-degradable polymers such as gelatin, gellan gum, etc. have attracted wider attention [8, 9]. While because these polymers are hydrophilic, the microspheres at wet state were soft with poor elasticity. In addition, the poor tough performance of these degradable polymer based microspheres at wet state make them easy to be break during operation [10, 11]. The soft and easily damaged features may cause the reduced embolization efficiency, leading to incomplete tumor embolism and necrosis. Many patients thus need repeated embolization because of the incomplete embolization. Of note, in routine clinical operation, reflux is the sign of embolic completion. To achieve enough embolization, extra amounts of embolic microspheres are often required during the repeated embolization, which may be accompanied by more microspheres reflux that would cause side effects. Developing embolic microspheres with higher embolization efficiency may reduce the dosage of embolic agents, significantly lowering side effects caused by reflux.

In our daily life, we can find that cork for red wine can fully block the wine liquid from leakage. And in laboratories, rubber stopper with high elasticity and toughness also effectively block the glass bottle mouth. These materials possess good deformation ability to adapt the size of glass bottle, and strong modulus for effective blocking. This reminds us that it is valuable to carry out an attempt of fabricating tough and elastic microspheric gel with stronger modulus for higher embolization efficiency. We assume in the present study that toughness makes the embolic agent difficult to be damaged, while strength and elasticity enable it to exert sufficient reverse force on embolized vessels, so to realized efficient plugging.

Simultaneously improving strength and toughness of hydrogel still remains a challenge [12]. Among various strategies to fabricate strong and tough hydrogels, developing double-network (DN) hydrogels networks that combine two networks with heterogeneous structure and complementary properties are denoted to be an effective strategy to prepare hydrogels with enhanced mechanical performance [12–15]. Furthermore, reversible non-covalent interactions are key factors to realize the improvement of both strength and toughness. For one thing, although non-covalent bond is weaker than covalent bond, polymer network with massive non-covalent interactions can still exhibit stronger mechanical behavior. For another, reversible non-covalent interactions can dissipate stress by fracture and formation, effectively promoting toughness and elasticity at the same time [15–18].

**Figure 1.**
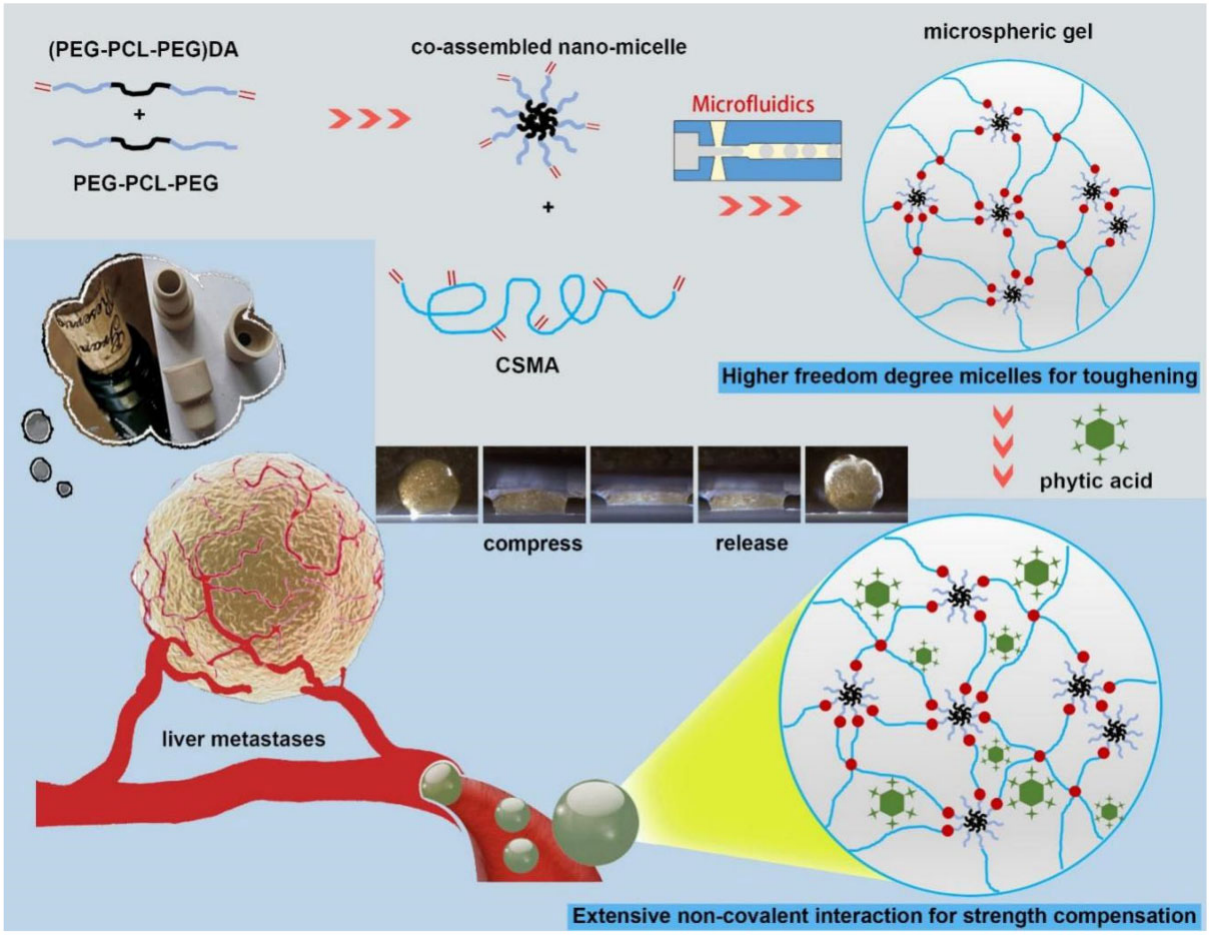
Schematic illustrations of the tough and elastic microspheric gel for chemoembolization.

Connecting micelles into hydrogel network is one of the DN derivatives. Non-covalent interactions in micelles take the responsibility to consume the external loading via its dissociation, making hydrogel withstand large deformation [19, 20]. In addition, the micelles also can work as carriers of drugs to realize continuous release [21]. Thus, inspired by rubber plugs, the present study developed a tough and elastic microspheric gel with stronger modulus via a modified micelle-based DN method, with further assistance of biogenic molecules, for chemoembolization in treatment of liver metastasis tumor. Amphipathic triblock copolymer poly(ethylene glycol)-*co*-poly(ε-caprolactone)-*co*-poly(ethylene glycol) (PEG-PCL-PEG, PPP), and PEG-PCL-PEG with two terminal double bonds ((PEG-PCL-PEG)DA, PPPDA) were co-assembled to form nano-micelles, which were bonded to methacrylated chitosan (CSMA) to fabricate microspheric gels with controllable diameter and high toughness. The microspheric gels were further non-covalently cross-linked with phytic acid (PA) to realize tough, strong and elastic microspheric gels. Then, a rabbit VX2 liver metastasis tumor model was established to evaluate the embolic effect of these microspheric gels, and compared them with commercially available embolic microspheres that have been used clinically (Fig. 1).

## Experimental section

### Fabrication of micelles

Poly (ethylene glycol)-*co*-poly(ε-caprolactone)-*co*-poly(ethylene glycol) (PEG-PCL-PEG, PPP; PEG, *M*_n_=2000 Da; PCL, *M*_n_=2000 Da) was purchased from Shanghai Jingchen Biotechnology Co., Ltd. PEG-PCL-PEG with two terminal double bonds ((PEG-PCL-PEG)DA, PPPDA) were synthesized according to our previous work [22]. Its ^1^H NMR spectrum was showed in the Supplementary Fig. S1. PPP and PPPDA were dissolved in deionized H_2_O with polymer concentration of 0.1 mg/ml and treated with ultrasonic wave for 30 min to yield micelle solutions. TEM and DLS were carried out to measure the morphology and size of different micelles.

### Fabrication of microspheric gel

Microfluidic devices consisted of two side inlets for oil flow (70 µl/min), one center inlet for polymer aqueous solution flow, and one outlet for droplets. Mineral oil supplemented with span 80 (2 w/w%) and CSMA solutions (1.5 w/v%, CSMA with double bond modification rate of 50%, *M*_v_=200 kDa, purchased from EFL) or CSMA-micelles solutions (3 w/v%; CSMA 1.5 w/v% (CSMA with double bond modification rate of 50%, purchased from EFL); micelles 1.5 w/v%) with lithium phenyl-2,4,6-trimethylbenzoylphosphinate (LAP, 0.1 w/v%, purchased from EFL) were pumped into the microfluidic devices. The water-in-oil droplets were generated at the junction of the four micro-channels. The droplets were crosslinked under UV irradiation (320–390 nm, 200 W/cm^2^, 15 min) to yield microspheric gels. The produced microspheric gels were observed and dimension-measured (V_1_) by stereomicroscope, followed by being immersed in PBS for 24 h for dimension-measurement (V_2_) to calculate swelling volume ratio. The swelling volume ratio was calculated according to the following formula: Sv=(*V*_2_−*V*_1_)/*V*_1_. For the swelling mass ratio test, dried microspheric gels were weighed (M_0_) after frozen at −80°C and lyophilized. They were immersed in PBS for 12 h to fully swell, and then centrifuged at 12 000 rpm for 10 min to remove the supernatant. The fully swollen microspheric gels were weighed (M_1_). The swelling mass ratio was calculated according to the following formula: Sm=(*M*_1_−*M*_0_)/*M*_0_. Microspheric gels were immersed in PA solution with different concentration for further enhancement. Fourier transform infrared (FT-IR) measurements were performed with an AVATAR 370 FTIR spectrophotometer from Nicolet.

### Mechanical tests on bulk hydrogels

Given the limitation of mechanical test on microspheres, the present study fabricated bulk hydrogels and hydrogel strips to illustrate their mechanical performance. The preparation method and component of bulk hydrogel were similar to those of microspheric gels. The compressive and tensile tests were performed at room temperature on an Instron 5943 testing machine. Compression tests were carried out on cylindrical samples with a diameter of 8 mm and height of 4 mm at 10% strain per minute and stopped at 90% strain. Tensile tests were carried out on dumbbell-shaped samples (2 × 1.5 mm) with a gauge length of 10 mm at a stretching crosshead speed of 100 mm/min. To clearly show the fine strip after stretching, the hydrogel strips used for observation in tensile test were all strained with Acid Black.

### Evaluation of blocking effect

The capillary glass tube with inner diameter of 300 μm was immersed in gelatin solution (5 w/v%, purchased from EFL) and vacuum dried. A 3D printing equipment (EFL BP6602 Pro, China) was employed for its pneumatic extrusion device, which was linked to the gelatin coating capillary glass tube hermetically. Microspheric gels with diameter of 500 μm were delivered into the capillary glass tube. The equipment started to start pressurization. The minimum air pressure required to start the movement of microspheric gels was observed and recorded. At the same time, the moving speed was evaluated by monitoring the time required for microspheric gels to move 10 cm.

### Evaluation of biocompatibility

The effect of microspheric gels on cell survival was studied using a live/dead stain assay. At 3 and 7 days, fibroblasts co-cultured with microspheric gels (5 mg and 10 mg) were stained with live-dead cell staining kit (Invitrogen). Briefly, cells were rinsed with PBS and incubated in staining solution at 37°C for 30 min followed by washing with PBS. Then cells were visualized via fluorescence microscope. Healthy cells fluoresced green, while the nucleus of dead cells appeared red.

The relative cytotoxicity was evaluated by Cell Counting Kit-8 (CCK-8). Fibroblasts were seeded in 96-well plates (10 000 cells per well) for 24 h, followed by adding microspheric gels (5 mg and 10 mg) for incubation for another 48 h. Then, CCK-8 solution (10 μl) was added to each well of the plate to be incubated for 4 h, followed by measuring the absorbance (optical density (OD) value) at 450 nm using a microplate reader (SpectraMax M2, Molecular Devices).

### Evaluation of degradation *in vivo*

To evaluate the degradation of microspheric gels, microspheric gels (10 μl) were implanted subcutaneously into C57 mice. The degradation of microspheric gels was observed on Days 14 and 28 after treatment. Then, the samples were collected, fixed with 4% paraformaldehyde, and sliced with paraffin-embedded tissue for H&E staining.

### Preparation of rabbit VX2 liver metastasis tumor model

Fifty adult New Zealand domestic rabbits, female, normal grade, four months old, weighing 2.2–2.5 kg, purchased from Shanghai Chendun Animal Co., Ltd, were kept by the Experimental Animal Experiment Center of Shanghai University of Traditional Chinese Medicine, license number: SYXK (Shanghai) 2020-0009. The animal study was approved by the Ethics Committee of the Animal Experiment Center of Shanghai University of Traditional Chinese Medicine (No. PZSHUTCM210115018). All animals were kept in standard rabbit hutches, with a standard room temperature of 25°C and a humidity of 60%, 12 h of light and 8 h of radiation per day to maintain a hygienic living environment. VX2 tumor strain was donated by Jinxia Mi’s group at the Animal Experiment Center of Shanghai University of Traditional Chinese Medicine.

VX2 solid tumor cell strain grew in New Zealand White rabbits to prepare a subcutaneous tumor model. Rabbit of the VX2 breeder was anesthetized by intramuscular injection of tiletamine hydrochloride 1:1 mixed with xylazine hydrochloride (0.2 ml/kg), and lied on the operating table with the tumor area on a hairless lateral position. The tumors were removed by clipping the skin and separating the subcutaneous tissue from the tumor. The tofu-like necrotic areas were removed from the tumor tissue, and the fish-like growth of the active tumor tissue was placed in a flat dish with an appropriate amount of PBS solution, which was then placed on an ice–water mixture. The tumor block was cut into a homogenate with ophthalmic scissors with addition of 10 ml PBS. The tissue homogenate was aspirated with a 20 ml syringe, and filtered through a 200 mesh cell strainer into an ice-pre-cooled test tube, which was centrifuged at 1500 r/min for 5 min to remove the supernatant. PBS solution was added into the test tube and filtered through a 300 mesh cell strainer to remove the cell clumps. The cells were counted, and the cell concentration was adjusted to 1 × 10^6/^ml–5 × 10^6^/ml, stored at 4°C.

Fifty New Zealand rabbits were selected, fasted for at least eight hours before implantation, anesthetized by intramuscular injection of tiletamine hydrochloride 1:1 mixed with xylazine hydrochloride (0.2 ml/kg), tied in the supine position with the abdomen hairless on the operating table, sterilized, toweled, separated 5–8 cm along the right subcostal margin in an oblique incision, layer by layer until the abdominal organs were exposed to find the left mesentery. The spleen was exposed along the mesentery and then freed from the abdominal cavity by gentle traction.

A syringe containing 1 ml of VX2 tumor strain suspension was inserted obliquely along the thickest part of the spleen, and ∼0.3 ml of suspension was injected. The puncture site was quickly compressed with an appropriate amount of gelatin sponge block for 1–2 min to confirm that there was no bleeding and no overflow of suspension before the spleen was incorporated back into the abdominal cavity. Approximately 5 ml of the solution containing levofloxacin 0.9% sodium chloride was flushed into the abdominal cavity and then sutured layer by layer. The rabbits were treated with anti-inflammatory therapy for 3 days continuously after suturing to prevent postoperative infection.

After modeling, three rabbits died due to anesthesia, and two rabbits were not found to have liver metastases under CT. Forty-five rabbits were randomly divided into three groups: control group (*n* = 15), experimental microsphere group (*n* = 15) and control microsphere group (*n* = 15).

Seven-day post-operation, the rabbits were euthanized and dissected along the abdominal midline, tissues of the liver were used to determine the tumor location. The maximum long diameter (*a*) and short diameter (*b*) of the tumor were measured, and the total tumor volume (CV) was calculated according to *V*=(1/2) *ab*2. The long diameter (*a*) and short diameter (*b*) of the section necrotic area were measured in each group. Tumor growth rate and tumor necrosis rate were calculated 7 days after operation. Tumor growth rate=postoperative tumor volume/preoperative tumor volume×100%, tumor necrosis rate=necrotic area/tumor area×100%.

### Interventional technique

After anesthesia, surgical access to the femoral artery was performed, and a 3-F catheter (J-Glidecath, Terumo, Tokyo, Japan) was used to engage the celiac axis with fluoroscopic guidance (Discovery IGS 730, GE). Left hepatic artery was selected for catheterization. The tumor was identified on digital subtraction angiography as a region of hypervascular blush, and the different treatment regimens were administered with real-time fluoroscopic guidance to prevent nontarget delivery (an interventional radiologist with 8 years of experience). CS-PPPDA&PPP-PA microspheric gels (Experimental Microspheres, 50 w/w% microspheres with diameter of 50 μm, 50 w/w% microspheres with diameter of 100 μm) dispersed in PBS and commercial embolic microspheres dispersed in medium (Control Microspheres; Embosphere, S120GH, Merit Medical, diameter range of 40–120 μm according to instructions) were mixed with iodixanol injection for embolism. To quantitatively describe embolization efficiency, Experimental Microspheres dispersed in PBS were controlled to have similar density with Control Microspheres in its own medium. They were pushed into the blood vessels associated with iodixanol for 0.5 ml each time until complete stasis or non-target embolization was observed, which indicated embolism was achieved. The femoral artery was ligated, and the surgical cut-down was closed. Quantitative description of embolization efficiency in the present study was evaluated by the total volume of embolic agent used for successful embolism.

### Quantitative image analysis

CT plain tumor morphology was performed on 14 days after mold arization. After anesthesia, the rabbits were placed on the CT examination bed, and the abdominal band pressure was controlled by breathing amplitude. The required tube voltage, tube current, scanning visual field (field of view, FOV) were 120 kV, 250 mA and 200 mm, and the scanning layer thickness was 2.5 mm. All images were reconstructed with a 1 mm layer of thickness. The CT criteria for HCC (Hepatocellular Carcinoma) diagnosis were plain scan.

### Morphological observation

Euthanasia (with Xylazine Hydrochloride Injection) was performed. The liver, lungs, gastrointestinal tract, peritoneum, and kidneys were examined. Fresh samples were taken from tumor, liver parenchyma around the tumor, and liver parenchyma (median lobe). Samples were fixed in 4% formalin for histopathologic examination. To evaluate the systemic effects of microspheres, the non-necrotic region and the surrounding hepatic tissues were obtained from the peripheral tumor, sectioned at 5 mm stained with hematoxylin and eosin (H&E) for routine evaluation of histology and inflammation. HIF-1α, IL-10, IL-6, and TGF-β1 protein expression were detected by immunohistochemical assay. Images were further analysed by Image J to quantitatively compare HIF-1α, IL-10 IL-6, and TGF-β1 protein expression.

### Relative gene expression of tissues

Total RNA was isolated from tissues by using Trizol and determined from the optical absorbance at 260 nm. cDNA was synthesized from RNA using a PrimeScript 1st strand cDNA synthesis Kit (TaKaRa). Real-time polymerase chain reaction (RT-PCR) was carried out by using SYBRGreen PCR MasterMix in each reaction. Expression of HIF-1α, IL-10, IL-6, and TGF-β1 genes were detected. Primer sequences: rabbit GAPDH: 5′-CTTTGGTATCGTGGAAGGACTC-3′ & 5′-GTAGAGGCAGGGATGATGTTCT-3′; HIF-1α: 5′-CTGTGATGAGGCTTACCATCACG-3′ and 5′-CTCGGCTAGTTAGGGTACACTTC-3′; IL-10: 5′-GAGAAGCATGGCCCAGAAATC-3′ and 5′-GAGAAATCGATGACAGCGCC-3′; IL-6: 5′-ATAGTCCTTCCTACCCCAATTTCC-3′ and 5′-GATGAATTGGATGGTCTTGGTCC-3′; TGF-β1: 5′-CAGTACAGCAAGGTCCTTGC-3′ and 5′-ACGTAGTAGACGATGGGCAG-3′. Relative expression levels for each gene were calculated by normalizing the quantified cDNA transcript level to that of the GAPDH.

### Statistical analysis

Each measurement reported was based on duplicate analysis of at least three independent experiments. The quantitative results were presented as mean±SD. One-way analysis of variance (ANOVA) was used to reveal statistical differences, and *P* < 0.05 was considered statistically significant.

## Results and discussion

### Bulk hydrogels preparation and mechanical performance enhancement evaluation

In order to develop a suitable hydrogel mechanical enhancement strategy and preparation parameters, bulk hydrogels were prepared firstly to clearly show the mechanical performance of the hydrogels with different networks.

**Figure 2.**
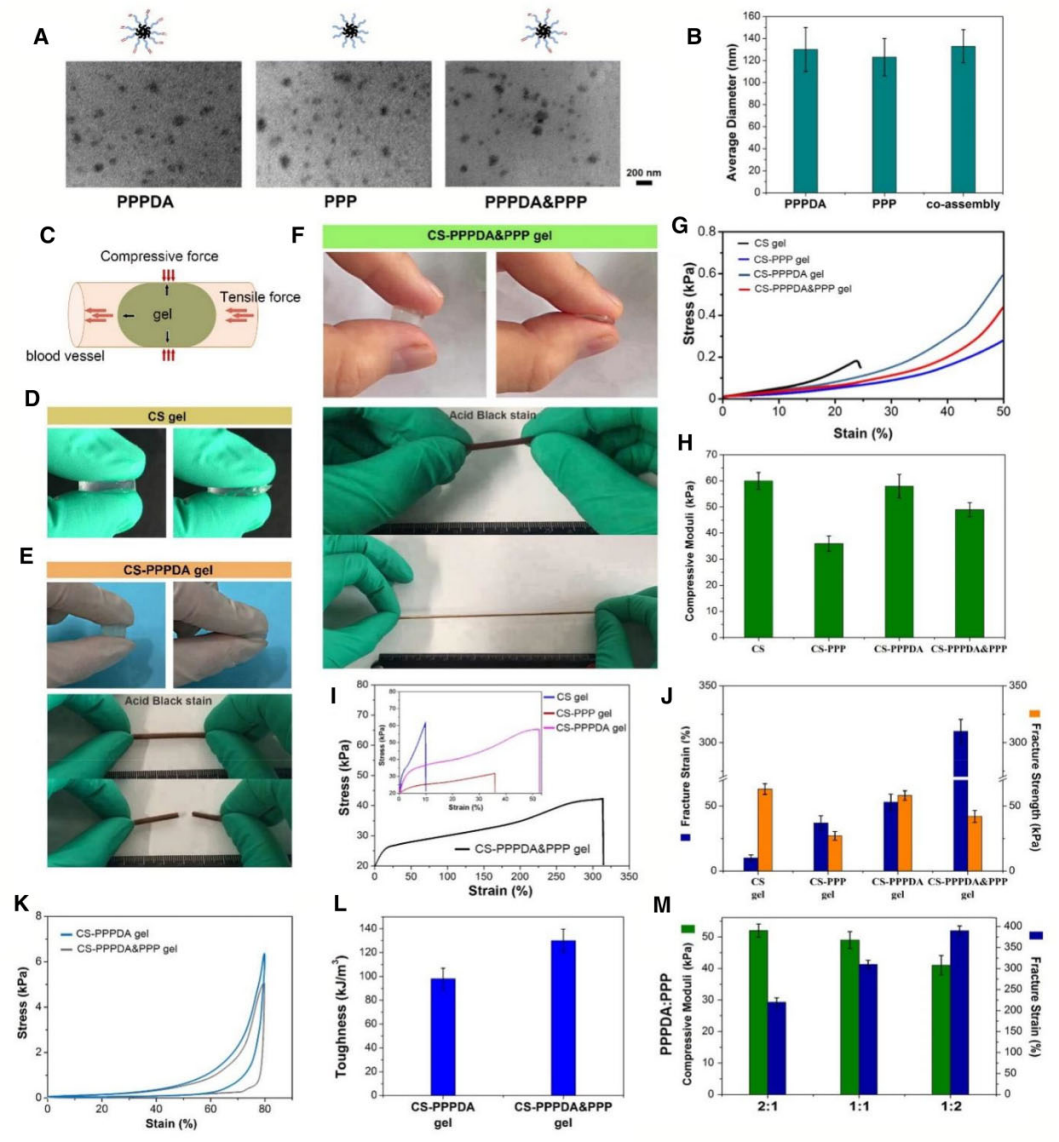
Preparation, compression and tensile testes of bulk hydrogels. **(A)**TEM images of PPP micelle, PPPDA micelle and co-assembled PPPDA&PPP micelle (bar scale: 200 nm). **(B)** Average particle size statistics. **(C)** Diagram to illustrate the research focus on strength and toughness of hydrogel. **(D)** General observation of CS gel under compressive force. **(E)** General observation of CS-PPPDA gel under compressive force and tensile force. **(F)** General observation of CS-PPPDA&PPP gel under compressive force and tensile force. **(G)** Stress–strain curves from compression test. **(H)** Compressive moduli. **(I)** Stress–strain curves of hydrogel strip under tensile force. **(J)** Fracture strain and fracture strength. **(K)** Loading–unloading compression tests. **(L)** The corresponding calculated toughness of CS-PPPDA gel and CS-PPPDA&PPP gel. **(M)** Compressive moduli and fracture strain of CS-PPPDA&PPP gel with different ratio of PPPDA and PPP. After the introduction of PPPDA micelles into CS hydrogel, it was found that the CS-PPPDA gel showed improved toughness. Under the action of compressive force, the hydrogel could deform larger (Fig. 2E). upon loading, the micelles were able to deform and even detach to dissipate energy. Due to the limitation of compression distance, the hydrogels were fabricated into strip for tensile test, which, however, showed that the CS-PPPDA gel was still easily destroyed. PCL is a hydrophobic polymer. In water, PCL segments in PPPDA chains were forced to aggregate in the core of micelles[21]. the cohesive force of PCL in water endowed the hydrogel with excess physical crosslinking units. Together with double bonds on micelle surface, the CS-PPPDA gel (mass ratio of CS/PPPDA was 1:1) possessed higher compressive moduli than other gels (Fig. 2G and H). In such case, the micelles acted as the huge crosslinking units that tightly bound inside gel network, indicating that PPPDA molecules that made up the micelles were covalently connected with CS and with each other. Therefore, the degree of micelle freedom decreased. According to Fig. 2I and J, although the fracture strain of CS-PPPDA gel was significantly improved when compared with CS gel, the improvement was limited.

At first, three kinds of micelles were fabricated from poly(ethylene glycol)-*co*-poly(ε-caprolactone)-*co*-poly(ethylene glycol) (PEG-PCL-PEG, PPP) and PEG-PCL-PEG with two terminal double bonds ((PEG-PCL-PEG)DA, PPPDA), including PPP micelle, PPPDA micelle and co-assembled PPPDA&PPP micelle. The two amphiphilic block polymers in aqueous solution possessed self-assembly behavior that driven by hydrophilic and hydrophobic interactions. The hydrophobic PCL formed the core while the hydrophilic PEG chains form the shell [22]. According to Transmission Electron Microscope (TEM) images in Fig. 2A, there were granular micelles that could be observed in the three groups. Through particle size statistics from Dynamic Light Scattering (DLS) tests, it was found that the three kinds of micelles showed similar average size, which was about 150 nm (Fig. 2B). Thus, the co-assembly of PPPDA&PPP did not significantly affect the micelle size. Then, bulk hydrogels were fabricated by mixing the CS-MA and different micelle solutions with the presence of the initiator of radical polymerization. Considering that the microgels embedded in blood vessel receive the tensile force caused by blood flow and the compressive force caused by vasoconstriction in the blood vessel, the compression and tensile properties were mainly focused (Fig. 2C). The compressive strength was related to the plugging efficiency, and the fracture strain was related to the toughness of microgel that resist to destruction. Besides, given the high viscosity, the maximum total polymer concentration in the present study was 3% (CSMA concentration was 1.5%) for ensuring the operability of microfluidic. At first, as shown in Fig. 2D, hydrogel fabricated from CSMA showed brittle feature similar to most single network hydrogels under compression, because natural biological macromolecules such as CS are hydrophilic, which can adsorb a large amount of water, leading to the expanding of network and decrease of network density. Introduction of other networks to restrict the hydrogel network from excessive expansion is an effective strategy to toughen hydrogel [10, 11].

To further improve the toughness of hydrogel, the present study proposed a modified micelle-toughening hydrogel strategy, which was to improve the degree of micelle freedom in hydrogel network. Thus, co-assembled PPPDA&PPP micelle was used to combine with CSMA to prepare hydrogel, CS-PPPDA&PPP gel. In this hydrogel, macromolecules that make up micelles partly chemically connected into the network, leaving other macromolecules freely embedded. Upon loading, the free macromolecules in micelles were able to move more freely with the sliding and deforming of micelles, delaying the dissociation of micelles. Thus, as shown in Fig. 2F, CS-PPPDA&PPP gel could not only be compressed to the limit without broken, but also perform significantly larger deformation in stretching test. The fracture strain of CS-PPPDA&PPP gel was about 300%, which was improved significantly when compared with CS-PPPDA gel (Fig. 2I and J). According to Fig. 2K and L, the area in the loading–unloading curve represents the energy dissipation of the hydrogel during compression tests, indicating the toughness of hydrogels. The energy dissipation of the CS-PPPDA&PPP gel hydrogel increased significantly when compared with CS-PPPDA gel, illustrating that the toughness of hydrogel was further improved by increasing the freedom degree of micelles in hydrogel. However, due to the loss of chemical crosslinking density, the compressive modulus of CS-PPPDA&PPP gel decreased (Fig. 2G and H). Thus, this strategy could significantly promote the toughness of hydrogel, while losing the strength. Specifically, according to Fig. 2M, the effect of mass ratio of PPPDA and PPP on mechanical performance was evaluated, showing that higher content of PPP led to decrease of compressive modulus and increase of fracture strain.

**Figure 3.**
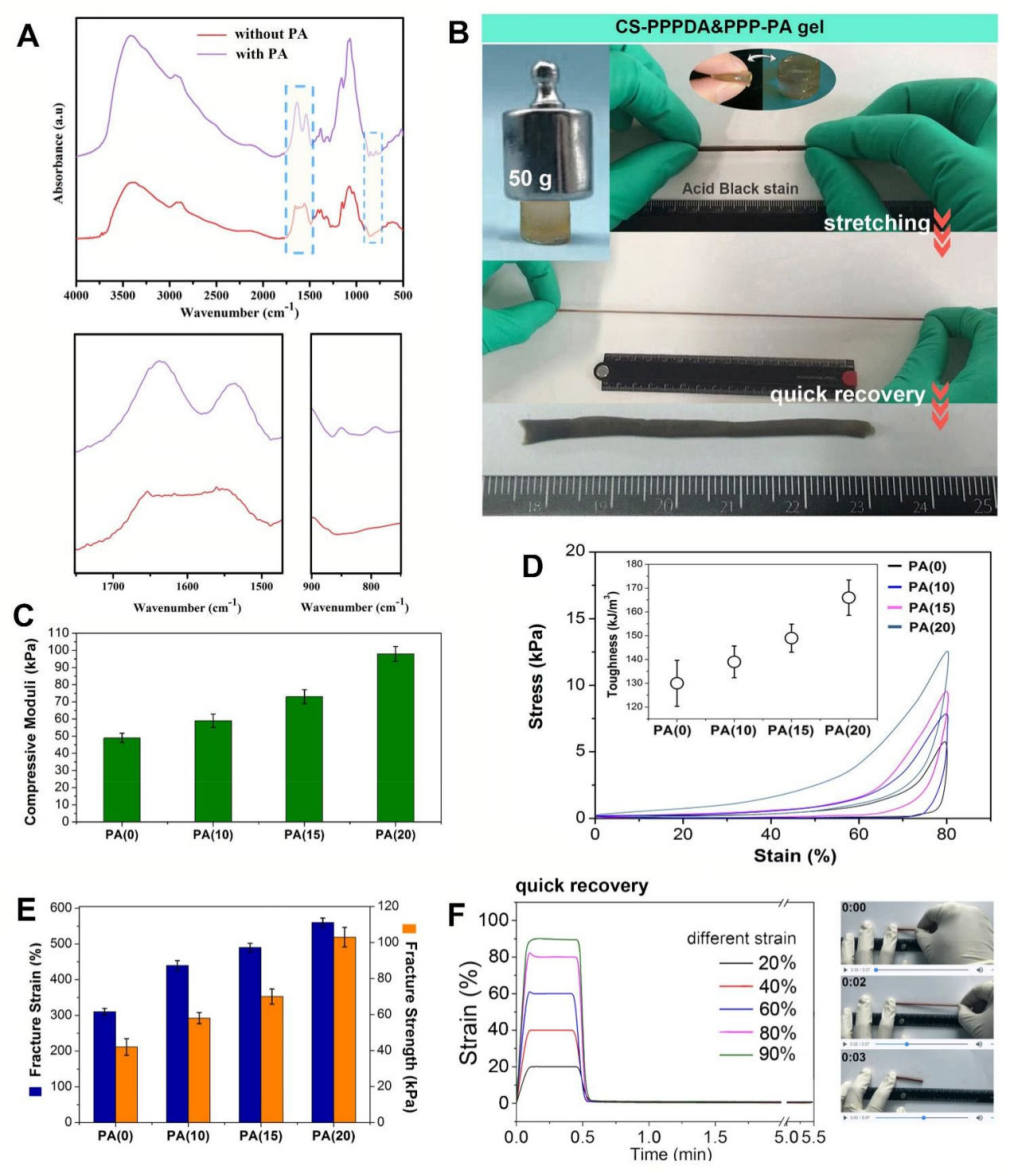
Preparation and mechanical performance CS-PPPDA&PPP-PA gels. **(A)** FT-IR spectra to show the interactions between CS and PA. **(B)** General observation of strong, tough and elastic CS-PPPDA&PPP-PA gel. **(C)** Compressive strength of CS-PPPDA&PPP-PA gel with different PA concentration. **(D)** Loading–unloading compression tests and the corresponding calculated toughness. **(E)** Fracture strain of CS-PPPDA&PPP-PA gel with different PA concentration. **(F)** Elasticity tests.

Phytic acid (PA), a biogenic molecule, which has six phosphate groups, was employed to interact with a dozen of amino groups of CS to form a compact physical network, so to further compensate for strength losses [12]. The interaction between CS and PA was evaluated by FT-IR. The presence of band at 1630 and 1530 cm^−1^ was inferred to the binding of PA to CS. In addition, shallow peaks at 845 and 795 cm^−1^ attributed to the P-H groups of PA were observed, further confirming the intermolecular interactions (Fig. 3A). The CS-PPPDA&PPP gel immersed in PA solution was defined as CS-PPPDA&PPP-PA gel, which showed significantly enhanced mechanical strength as shown in Fig. 3B. At the same time, the toughness was further improved. The hydrogel strip exhibited excellent flexibility and could withstand high-level deformation of elongation, and return to its original form quickly. As shown in Fig. 3C, the compressive modulus increased with the increase of PA concentration. And according to compressive loading–unloading curves (Fig. 3D), the toughness of CS-PPPDA&PPP-PA gel further improved with the increase of PA concentration. This was related to physical interaction between PA and CS, which might be used as sacrificial interactions to dissipate stress. In addition to compression tests, tensile tests further confirmed that the introduction of extensive non-covalent interaction into hydrogel guaranteed the improvement of both strength and toughness (Fig. 3E). Moreover, recovery tests showed that hydrogel with strain from 20% to 90% could recover immediately after force removal (Fig. 3F). The fast recovery after deformation indicated the well-performed elasticity.

### Preparation of microspheric gels and blocking effect evaluation

**Figure 4.**
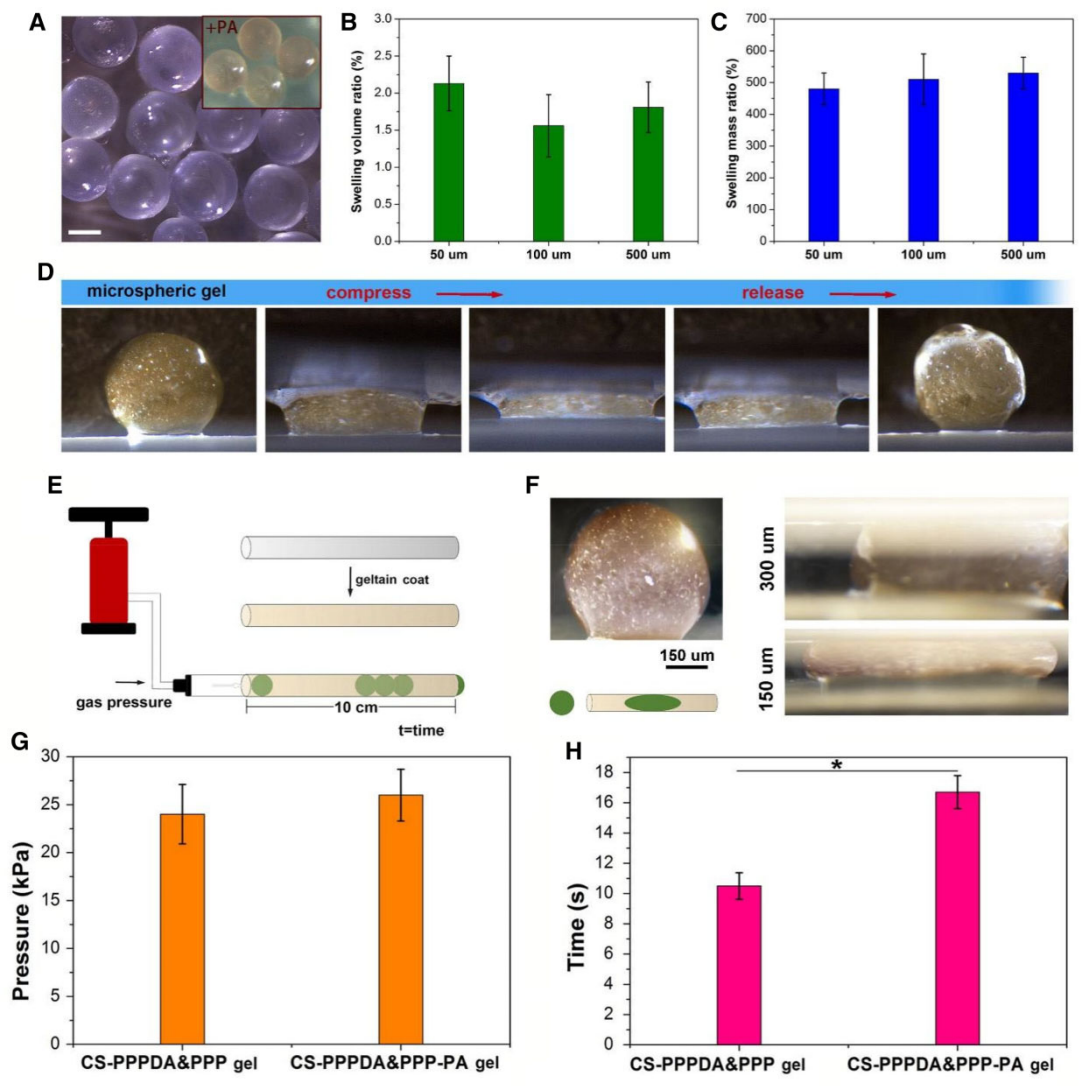
Preparation and evaluation of microspheric gels. **(A)** Microspheric gels produced from microfluidic device in PBS observed by stereomicroscope (bar scale: 50 μm). **(B)** Volume change of microgels produced directly from the microfluidic equipment after being immersed in PBS for 12 h. **(C)** Maximum equilibrium swelling degree. **(D)** Compression test on CS-PPPDA&PPP-PA microspheric gel with the diameter of 1000 μm to show its toughness and elasticity. **(E)** Diagram to illustrate the blocking test. **(F)** Microscopic observation of microspheric gel with diameter of 500 μm inserted into capillaries with diameter of 300 μm and 150 μm. **(G)** Minimum air pressure to move the microspheric gels. **(H)** Time required for microspheric gels to move 10 cm under minimum air pressure (*n* = 5, *P < 0.05).

With the assistance of microfluidic technology, microspheric gels were fabricated by mixing the CS-MA and PPPDA&PPP micelle solutions with the presence of the initiator of radical polymerization. The prepared microspheric gels were then immersed in PA solution with the concentration of 10%. The diameter of microspheric gels could be controlled via changing the size of microchannels. In the present study, we created microspheric gels with the diameter of 50, 100 and 500 μm. As shown in Fig. 4A, microgels showed relatively regular spherical shapes. For one thing, the microgels produced directly from the microfluidic equipment showed insignificant volume change after being immersed in PBS, indicating the formed network was strong enough to resist excessive swelling (Fig. 4B). For another, the microgels with each size showed maximum equilibrium swelling degree of about 500% (Fig. 4C). A CS-PPPDA&PPP-PA microspheric gel with the diameter of 1000 μm was prepared to show its toughness and elasticity. As shown in Fig. 4D, the microspheric gel could withstand large deformation of compression and recover quickly.

To evaluate the blocking effect of microspheric gels, a gelatin-coated capillary glass tube with inner diameter of 300 μm was hermetically linked to the pneumatic nozzle, which was used to deliver microspheric gel with diameter of 500 μm and record pressure data (Fig. 4E). The minimum air pressure to move the microspheric gels, as well as the time required for microspheric gels to move 10 cm was monitored. At first, the microspheric gel that inserted into the capillary was observed with the assistance of microscope. Because the inner diameter of the capillary was smaller than the microspheric gel diameter, the microspheric gel inserted in capillary was condensed significantly, showing a stretched state with extended long-axis length. In addition, if the microspheric gel was inserted into a capillary with inner diameter of 150 μm, the microspheric gel was further condensed, exhibiting larger extension along with the capillary tube without damage, indicating the high toughness of the CS-PPPDA&PPP-PA(10) microspheric gel (Fig. 4F). We thus speculated that under such high degree of compression, the microspheric gel could storage of potential energy by deformation, producing strong reacting force on blood vessels to achieve high blocking effect. If the microparticles were soft or easily damaged, the potential energy generated by compression would be small or lost through the destruction of the materials. Moreover, as shown in Fig. 4G and H, the air pressure to move CS-PPPDA&PPP microspheric gels was similar to that of CS-PPPDA&PPP-PA(10) microspheric gels. However, under same air pressure, the moving speed of CS-PPPDA&PPP-PA(10) microspheric gels was significantly slower than that of CS-PPPDA&PPP microspheric gels, revealing that the CS-PPPDA&PPP-PA microspheric gels exhibited stronger blocking effect. Besides, spherical microgels are more likely to be trapped in the vasculature, which can help to block the flow of blood, so as to cause embolization. However, non-spherical particles may not be as effective at embolization, as they may not be able to effectively block the flow of blood [23].

### Evaluation of biocompatibility and degradability

**Figure 5.**
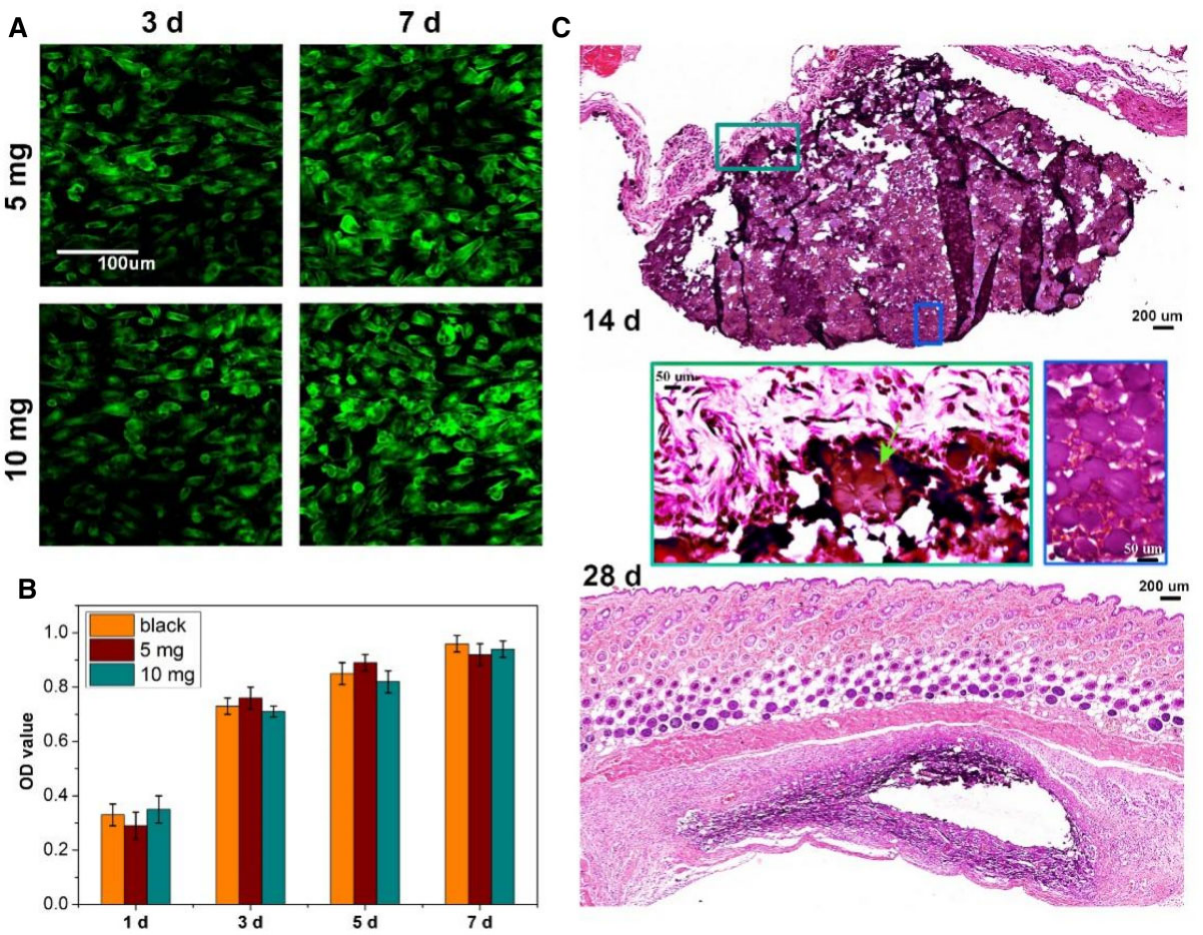
Biocompatibility and degradability of CS-PPPDA&PPP-PA(10) microspheric gels. **(A)** Representative fluorescence images observed by live/dead staining assay to show the viability of fibroblasts co-cultured with the microspheric gels. **(B)** CCK-8 assay to demonstrate the cytotoxicity of microspheric gels in cell proliferation (*n* = 5). **(C)** H&E staining of subcutaneously implanted microspheric gels at 14- and 28-day post-implantation (*n* = 3).

The biocompatibility of CS-PPPDA&PPP-PA(10) microspheric gels was evaluated further. As shown in Fig. 5A, after co-culturing with 5 mg and 10 mg microspheric gels, live/dead staining images showed that most fibroblasts exhibited favorable viability at 3 and 7 days. Dead cells (red staining) were hardly observed. CCK-8 assay further showed the number of live cells in the experimental groups was similar to the blank group, and there was no significant difference in cell proliferation among the three groups, indicating that the microspheric gels possessed biocompatibility and had no cytotoxicity (Fig. 5B). At the same time, *in vivo* degradation of microspheric gels was evaluated. Microspheric gels with the volume of 10 μl were subcutaneously implanted. As shown in Fig. 5C, after 14-day post-implantation, aggregated microspheric gels could be observed clearly. After 28-day post-implantation, no obvious microspheric gels could be found, indicating the significant degradation of CS-PPPDA&PPP-PA(10) microspheric gels. In fact, the degradation behavior of microspheric gels in blood vessels may vary from their subcutaneous counterparts due to different fluid environments, enzyme composition, and other factors [24]. The fluid environment in blood vessels is typically more turbulent and has a higher flow rate, which may affect the degradation rate of the microspheric gels. Additionally, the enzyme composition of the blood is also different that in the subcutaneous environment [25]. For example, the types of enzymes in the blood microenvironment are typically proteins that are involved in the breakdown of nutrients, such as proteases, lipases, and carbohydrases [26]. The types of enzymes in the subcutaneous microenvironment are typically enzymes that are involved in the synthesis of proteins, such as glycosyltransferases, kinases, and phosphatases. The differences may alter the degradation rate of the microspheric gels by either increasing or decreasing the rate. However, the results of subcutaneous degradation *in vivo* still confirmed that the microspheric gels prepared in this study could degrade step by step, and the degradation rate was not too fast to affect the embolization effect.

### Metastatic liver cancer modeling

**Figure 6.**
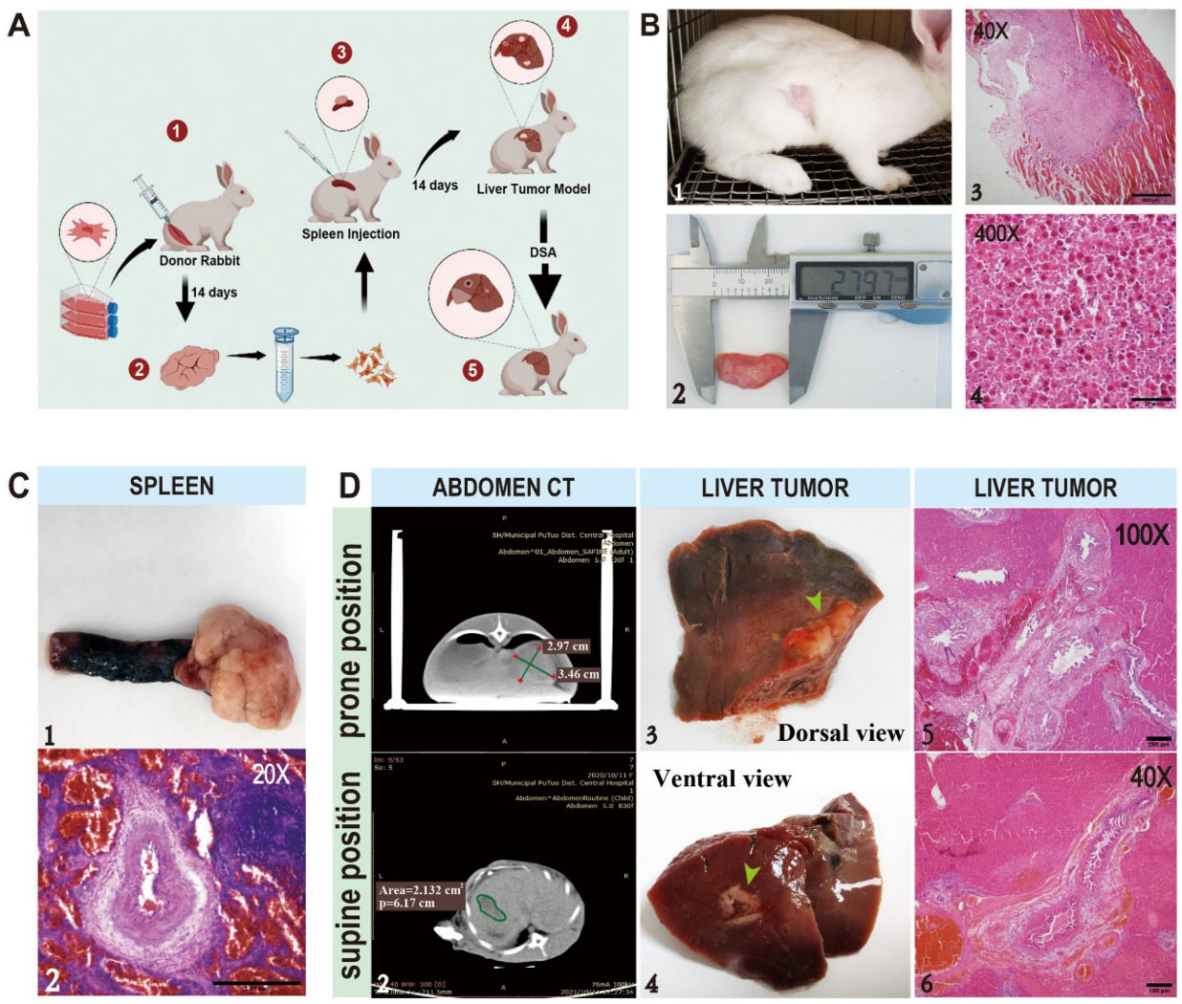
Metastatic liver cancer modeling. **(A)** Metastatic hepatocellular carcinoma modeling procedures. **(B)** The tumor-bearing rabbit model and H&E staining of the leiomyoma (bar scale: 500 μm for 40×, 20 μm for 400×). **(C)** Splenic tumor mass and H&E staining (bar scale: 3.45 mm). **(D)** CT image, general observation and H&E staining of the liver with occupied foci after 14 days (bar scale: 250 μm for 100×, 100 μm for 40×).

The liver metastasis tumor model was established by subsplenic capsule injection (Fig. 6A) [27]. As tumor-bearing rabbits, VX2 cells accompanied with myofiber cells were observed according to H&E staining. VX2 tumor cells clearly showed different morphology with myofiber cells, possessing less cytoplasm and deep nuclear staining (Fig. 6B). After the injection of VX2 cells into the spleen, the spleen tumor was successfully modeled as shown in Fig. 6C. The surface of the spleen was rough with an irregular mass grew at one end of the spleen. The cancer cells were diffusely distributed between the red and white marrow of the spleen in H&E staining image. Then, after 14 days, the metastasis in the liver region was clearly observed. A nodular hypodense lesion of ∼3 × 3 cm in the right hepatic region was observed on the plain scan of CT. Multiple metastatic lesions were also found on the dorsal and ventral view of the right lobe of liver. According to H&E staining, a large amount of cancerous tissue was observed to distribute diffusely with a solid adenoid arrangement of cancer cells, which were polygonal, cytoplasm-rich, granular, eosinophilic, with a large nucleoplasmic ratio and darkly stained nuclei (Fig. 6D). Before treatment, there was no significant difference in tumor volume between the two groups of model rabbits, indicating tumor model was consistent (Table 1).

**Table 1.** Comparison of tumor volume before treatment in three groups of rabbits under CT, (′*x* ± *s*, cm^3^)

Group	*n*	Volume
Control	15	1.44 ± 0.87
Experimental microspheres	15	1.25 ± 0.37
Control microspheres	15	1.39 ± 0.83
*F*		0.1875
*P*		0.8301

### Embolization in liver metastasis tumor

**Figure 7.**
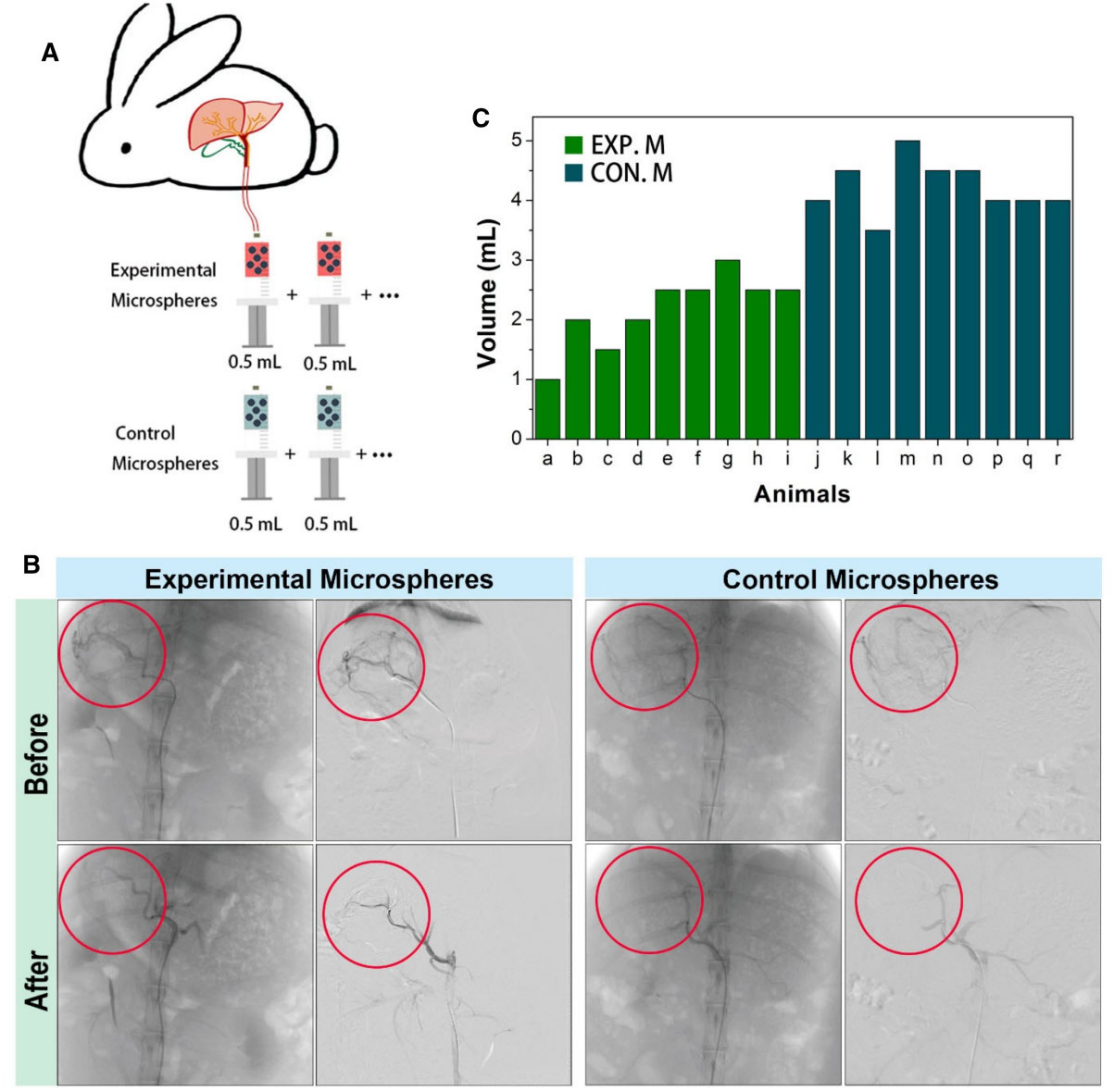
Embolization operation and statistics of embolic agent dosage during operation. **(A)** Diagram to illustrate the quantitative comparison method of intraoperative embolic agent dosage. **(B)** Before and after embolization of DSA images and subtraction images. **(C)** Volume statistics of applied embolic agent (*n* = 9).

Then, CS-PPPDA&PPP-PA microspheric gels (Experimental Microspheres) and commercial embolic microspheres (Control Microspheres; Embosphere, S120GH, Merit Medical) were used for embolism. Experimental Microspheres and Control Microspheres were dispersed in the contrast medium solution in a similar amount to prepare Embolic Agents. During interventional operation, 0.5 ml of Embolic Agent was injected each time until obvious reflux was observed. Total dosage of Embolic Agent was recorded to compare the embolic efficiency (Fig. 7A).

Under DSA, rabbit VX2 hepatocellular carcinoma were all supplied by the right hepatic artery. No other variants or occult supply arteries were observed. Following the injection of contrast, most tumors showed nodular staining. Some tumors showed circumferential staining at the edges and gradually populated towards the center. The terminal segment of the right hepatic artery and its branches were disturbed increasingly and encircled the tumors, showing the ‘ball-holding sign’. A mixture of Embolic Agent and iodixanol injection was injected into the right hepatic artery, showing significant vascular embolization and gradually deposition in the tumors. The embolization effect of the Experimental Microspheres was similar to that of the Control Microspheres under DSA (Fig. 7B). Quantitative description of embolization efficiency in the present study was evaluated by the volume of Embolic Agent used for successful embolism. According to Fig. 7C, to achieve reflux that indicated successful embolism, the volume of used Experimental Microspheres was lower than the volume of used Control Microspheres. Since the Experimental Microspheres dispersed in contrast medium solution possessed similar density with the Control Microspheres in medium, the lower volume indicated lower content of microspheres, thus higher embolization efficiency. By using fewer embolic agents, the potential risks of damage to surrounding tissue or organs would be reduced. The use of fewer embolic agents can reduce the risk of tissue injury and complications such as thromboembolic events and bleeding [28]. Additionally, it can reduce the amount of radiation exposure to the patient and reduce the amount of time it takes to complete the procedure [29]. On the other hand, it can reduce the cost of interventional surgery, as fewer embolic agents are required. In the current experiment, we have not encapsulated chemotherapy drugs or other anticancer drugs in the microspheres, but we have considered incorporating drug-carrying microspheres into the study scope in the future. This can help to reduce the side effects of the drug, as well as increase its efficacy.

**Figure 8.**
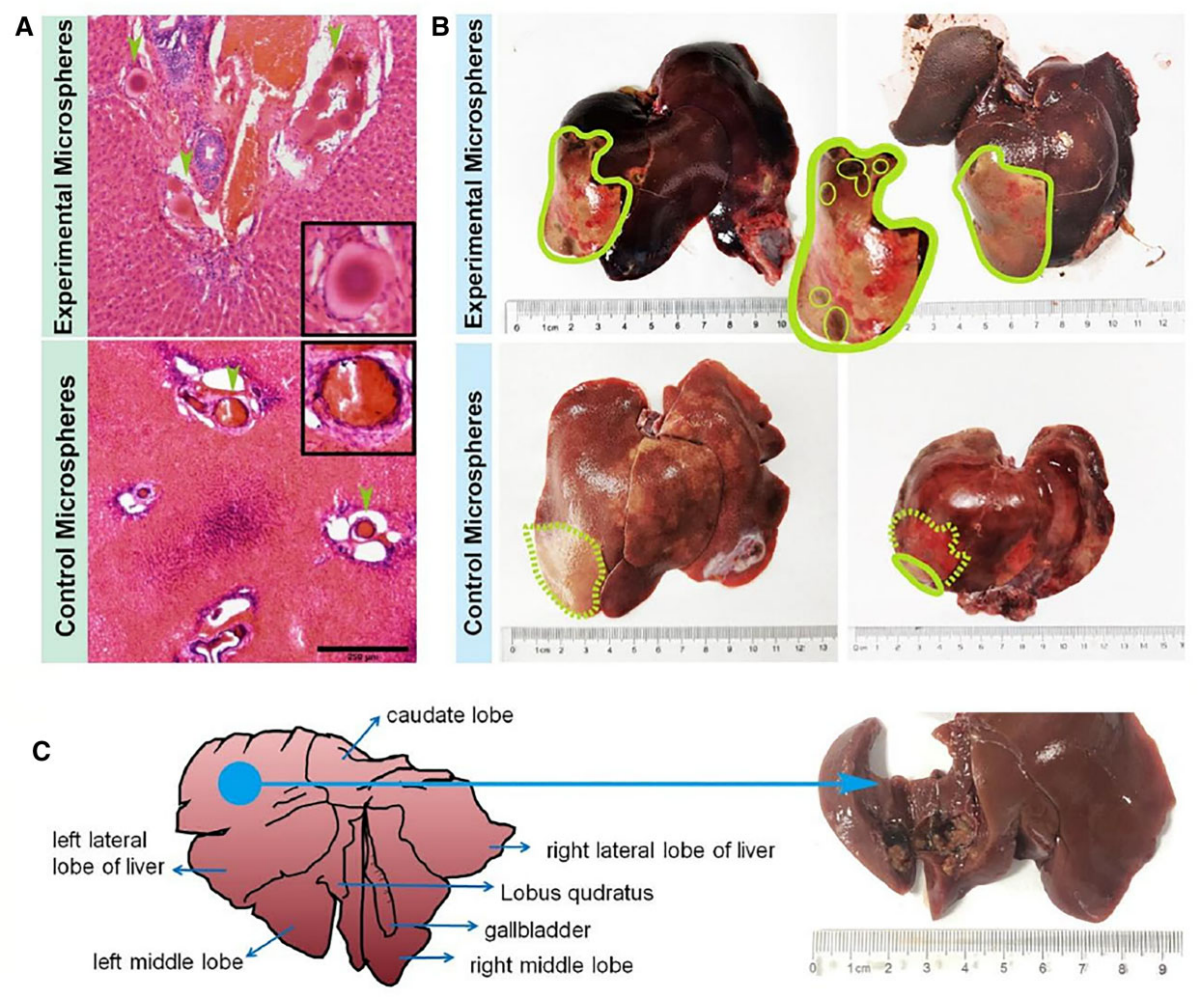
Effects of the interventional therapy. **(A)** H&E staining to show the microspheres accumulated in the blood vessels after embolization (bar scale: 250 μm). **(B)** General observation images of localized hepatic necrosis after DSA embolization. **(C)** The tumor tissue was dissected at the maximum diameter of the tumor to show complete tumor necrosis.

In addition, the Experimental Microspheres were scattered in the blood vessels after embolization, which showed regular morphology without damage. The surrounding cancer tissue was diffusely distributed with little interstitium. The hepatocellular carcinoma cells were arranged in a solid adenoid shape with a small amount of necrosis. Similarly, the Control Microspheres deposits were observed in the vessels. However, damage was found in some microspheres. The surrounding cancerous tissue was reduced to varying degrees and replaced by fibrosis. Necrosis was visible (Fig. 8A). The liver autopsy results after 14 days showed that both the Experimental Microspheres and the Control Microspheres had remarkable embolization effect with the presence of a blockage area, as shown in Fig. 8B. Besides, there were a few black abnormal spots in the necrotic area, which revealed necrotic tumor tissue. After embolization, necrosis was found at the original tumor mass in the liver, and legacy cavities were observed in Fig. 8C. After treatment, both groups of model rabbits experienced a significant reduction in tumor volume. While a lower tumor growth rate was found in the Experimental Microspheres treated group when compared with Control Microspheres treated group (Table 2).

**Table 2.** Tumor growth rate and number of metastases in different groups on Day 7 (%)

Group	*n*	Tumor growth rate (%)
Control	15	745.08 ± 146.04
Experimental microspheres	15	182.39 ± 70.47*
Control microspheres	15	209.73 ± 88.79*
*F*		87.15
*P*		0.00001

Results are in response to one-way analysis of variance. *P < 0.01: Control group compared with the other groups.

**Figure 9.**
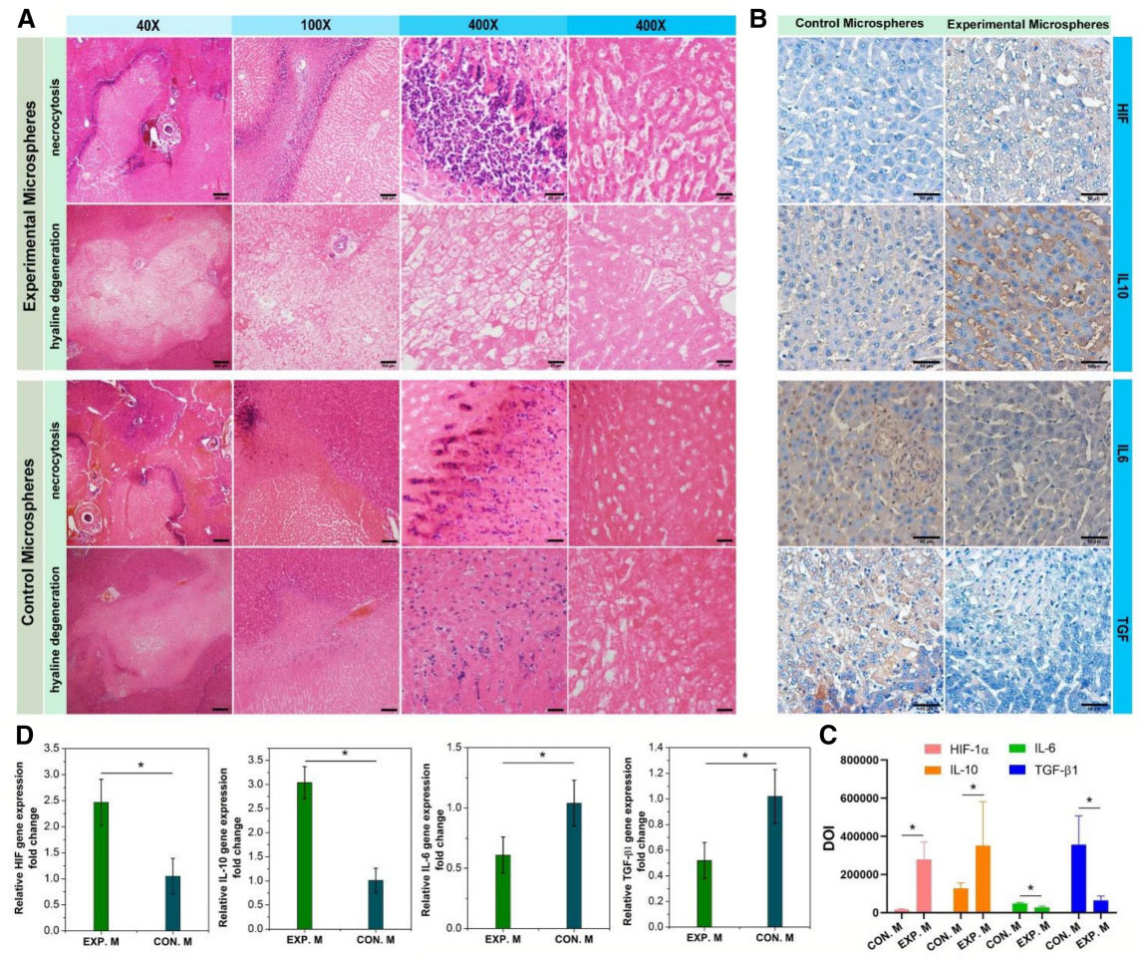
Effects of the interventional therapy. **(A)** H&E staining on liver sections isolated at 14 days after the treatment to show necrocytosis and hyaline degeneration (bar scale: 250 μm for 40×, 100 μm for 100×, 25 μm for 400×). **(B)** HIF-1α, IL-6, IL-10 and TGF-β1 immunohistochemical staining to demonstrate the microenvironment in the liver and tumor adjacent tissues at 14 days after the therapy to show embolic degree comparison between experimental microspheres and control microspheres (bar scale: 50 μm). **(C)** Image quantitative analysis from images in B (*P < 0.05). **(D)** Relative genes expression (*P < 0.05).

The area of the necrotic area of tumor tissue was observed further. The total number of nuclear divisions and cell morphology and structure were observed. The area of coagulated necrosis of the liver parenchyma showed swelling of the hepatocytes, nuclear consolidation and cytoplasmic light staining, widening of the hepatic sinusoidal space and congestion with a large number of erythrocytes accumulating in the sinusoids. At the same time, tissue was partially vacuolated due to hemoglobin loss, and a large infiltration of inflammatory cells around the necrotic area were observed (Fig. 9A). In addition, transparent hepatocellular degeneration, disorganized liver lobules and crowded hepatic cords were also observed. The hepatocytes were enlarged, and the hepatic sinusoids were distorted and narrowed. The cytoplasm of the hepatocytes was lax and hollow, reticulate or hyaline, with the nucleus suspended in the center and staining lightened. A portion of the hepatocytes was full and swollen with hyaline cytoplasm, forming a hyaline degeneration of the hepatocytes. H&E staining examination of group used Control Microspheres showed similar results with the group that treated with Experimental Microspheres. However, according to hyaline degeneration examination, it was found that the hyaline degeneration degree was lower, revealing that the embolic degree of Experimental Microspheres was higher.

Immunohistochemical staining and relative gene expression were carried out to reveal differences in the immunomodulatory effects of using different microspheres on the tumor immune microenvironment (Fig. 9B–D). Hypoxia is one of the key factors in regulating the development of various tumors, and one of the key pathogenic factors in determining the development and severity of the disease. The HIF-1α protein locates in the nucleus and cytoplasm, with the nucleus predominating, which is stained positively for pale yellow, yellow and brownish-yellow granules [30–33]. The group treated with Experimental Microspheres showed higher HIF-1α positive expression, revealing higher protein expression in the cytoplasm and nucleus. Hypoxia is one of the main effects of microsphere embolization, and it can induce the expression and activity of a transcription factor called HIF-1α in cells [34]. HIF-1 is a factor that has been widely studied, and it is one of the most common transcription factors in hypoxia [35]. Studies have shown that significant increase of HIF-1α expression is found in liver cancer cells after microsphere embolization, which may affect the occurrence and development of liver cancer [36]. It can promote the survival, proliferation and metastasis of tumor cells, and can affect the formation of tumor microenvironment. The activation of HIF-1 can also regulate the expression of many genes related to the occurrence and development of liver cancer, such as VEGF, PDGF, TGF-β etc. [37, 38], thereby promoting the growth and metastasis of liver cancer. However, according to the results, application of Experimental Microspheres with high embolic effect did show an up-regulated HIF-1α expression. Multi-functional microgels are generally used in clinical treatment. Due to the natural drug loading characteristics of the hydrogel network, and the loading and sustained release effect of micelles on hydrophobic drugs, the introduction of drugs into the present hydrogel microspheres may reduce the negative effects caused by the up-regulation of HIF-1α. The integration of multi-functional embolic microspheres will further optimize the therapeutic effect in future study.

At the same time, the group treated with Experimental Microspheres showed low expression of IL-6 and TGF-β1 and high expression of IL-10 than the group treated with Control Microspheres. IL-6, IL-10 and TGF-β1 were mainly designated in the cytoplasm and their positive expression was determined by the presence of brownish-yellow granules in the cell cytoplasm [39–41]. IL-6 is an immunoinflammatory cytokine which can activate the immune system after infection, thus promoting the secretion of IL-6 and exerting an immunoprotective effect [42, 43]. IL-10 can down-regulate the expression of pro-inflammatory factors, shows immunosuppressive effects, inhibits the expression of Th1 cytokines, and has anti-inflammatory effects [44, 45]. TGF-β1 has a systemic immunosuppressive effect and inhibits host immune surveillance. TGF-β1 also regulates the infiltration of inflammatory/immune cells and cancer-associated fibroblasts in the tumor microenvironment, leading to direct changes in tumor cells. TGF-β1 has adverse effects against tumor immunity and significantly suppresses host tumor immune surveillance [46–48]. The results illustrated that the use of Experimental Microspheres could reduce the expression of TGF-β1, improved the state of tumor immunosuppressive microenvironment. At the same time, use of Experimental Microspheres was more conducive to the recovery of local liver cancer after embolization, preventing and controlling the regeneration of tumor cells.

## Conclusion

In summary, aiming at the low embolic efficiency caused by soft and easily damaged features of embolic particles, the present study fabricated a tough and elastic microspheric gel for promoting embolic efficiency in TAE application. A strategy that increasing freedom degree of micelles plus non-covalent compensation to develop elastic and tough hydrogel was established. By introducing PEG-PCL-PEG & (PEG-PCL-PEG)DA co-assembled micelles into CSMA hydrogel, the present study significantly improved the toughness of hydrogel. By introducing PA, the strength and toughness of hydrogel were further enhanced, exhibiting high elasticity and tolerance to large deformation. Microspheric gels with the above network were produced via microfluidic devices, and showed higher blocking effect *in vitro*. *In vivo* evaluation further demonstrated that the microspheric gels embolization enabled hypoxia and necrosis of tumor target sites and paracancerous tissues while reducing inflammation. The higher expression of the hypoxic environment compared to the commercial microsphere indicated the long-term effectiveness of microsphere. In the process of interventional operation, the number of experimental microspheres required was small, which could not only reduce the intake of contrast media, but also ensure the embolization effect while reducing the risk of adverse effects on surrounding tissues.

## Supplementary Material

rbad026_Supplementary_Data
