# Supplementary material for: Highly tough and elastic microspheric gel for transarterial catheter embolization in treatment of liver metastasis tumor
Source: Regen Biomater. 2023 Mar 21;10:rbad026. doi: 10.1093/rb/rbad026 (PMC10067152; doi:10.1093/rb/rbad026)
Supplement: rbad026_Supplementary_Data [file rbad026_supplementary_data.docx]

**Highly tough and elastic microspheric gel for transarterial catheter embolization in treatment of liver metastasis tumor**

Shuyun Wang ^1,#^, Hongjie Yu ^1,#^, Guangsheng Wan ^1,#^, Haowei Fang ^3^, Jinxia Mi ^4^, Wenqian Xu ^1^, Kexiang Sun ^1^, Kunxi Zhang ^1,2,3,^*, Jingbo Yin ^3,^*, Wanli Deng ^1,^*

^1^ Department of Medical Oncology, Putuo Hospital, Shanghai University of Traditional Chinese Medicine, Shanghai, 200062, P.R. China.

^2^ Interventional Cancer Institute of Chinese Integrative Medicine, Putuo Hospital, Shanghai University of Traditional Chinese Medicine, Shanghai 200062, P.R. China.

^3^ Department of Polymer Materials, School of Materials Science and Engineering, Shanghai University, Shanghai 200444, P.R. China.

^4^ Research Center for Health and Nutrition, School of Public Health, Shanghai University of Traditional Chinese Medicine, Shanghai 201203, P.R. China.

^#^ These authors contributed equally to this work.

* Corresponding authors:

Kunxi Zhang, Email: zhangkunxi@shu.edu.cn

Jingbo Yin, Email: jbyin@oa.shu.edu.cn

Wanli Deng, Email: dwl0707@163.com


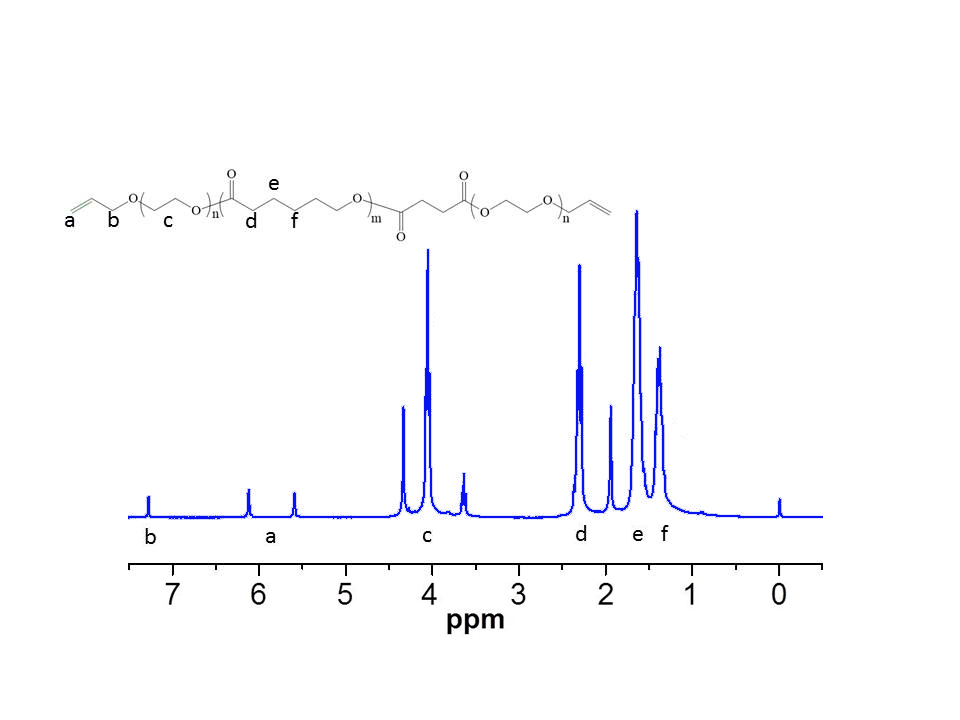


**Figure S1.** ^1^H NMR spectrum of (PEG-PCL-PEG)DA, PPPDA.
